# Graduate Study in London Special Hospitals

**Published:** 1917-09-08

**Authors:** 


### GRADUATE STUDY IN LONDON SPECIAL HOSPITALS.

**Central London Throat and Ear Hospital, Gray's Inn Road, W.C.**—Three courses of instruction are open to practitioners attending the hospital: first, the course in methods of examination and diagnosis; second, the course of systematic instruction in the diseases of the nose, throat, and ear; and third, the operative surgery class. The course in methods of examination and diagnosis is introductory in character. It comprises four lessons of practical teaching in the actual examination of patients and in the manipulation of instruments. Clinical assistants, especially if they have not had any previous experience in the speciality, are expected to attend this class in order to become acquainted with the minutiæ of the methods of diagnosis, etc. The class is free to clinical assistants. To ordinary students and practitioners the fee is one guinea. Intending students (who can join at any time) are requested to give their names to the Dean. Attendance at this class is compulsory for those who desire to obtain the higher-grade certificate. The second course of systematic instruction in diseases is more advanced. It consists of over thirty lessons in all on pathology, diagnosis, and treatment. Minute details in operative surgery are not gone into, as this is left to the operative surgery class. Full syllabus may be had on application to the Secretary.

**Hospital for Consumption and Diseases of the Chest, Brompton, S.W.**—Owing to the war the courses of special lectures usually given during term have been suspended, but the large clinical field of the hospital is still available for those who wish to study the diseases of the chest under the supervision of the physicians or assistant physicians. Students may enter for courses of clinical instruction in the wards or out-patient department at any time. Arrangements can be made for courses of instruction in special laboratory methods. Special courses of instruction in the diagnosis and treatment of pulmonary tuberculosis are held from time to time. For particulars apply to the Dean.

**Hospital for Diseases of the Throat, Golden Square, W.**—Clinical instruction in the diagnosis and treatment of disease is given daily in the out-patient department from 2 to 5 P.M., on Tuesdays and Fridays from 6.30 to 9 P.M., and on Mondays at 9.30 A.M. The hospital contains 60 beds for in-patients. There is an annual out-patient attendance of over 50,000. Minor operations are performed daily (except Monday) at 9.30 A.M. Major operations are performed on Tuesdays, Wednesdays, Thursdays, Fridays, and Saturdays at 10 A.M. Also Fridays at 2 P.M. Practitioners and medical students are admitted to the practice of the hospital at a fee of five guineas for three months, seven guineas for six months, or ten guineas for perpetual studentship. From amongst the students junior and clinical assistants are appointed periodically. Surgeons: Dr. J. W. Bond, Mr. Charles Parker, Mr. Jefferson Faulder, Mr. George Badgerow, and Mr. Patterson. Assistant Surgeons: Mr. Lionel Colledge and Mr. G. W. Dawson. Dental Surgeon: Mr. Pitts. Apply to Norman Patterson, Hon. Med. Sec.

**Hospital for Sick Children, Great Ormond Street, W.C.**—This school is one of the post-graduate teaching schools of the University of London, and is recognised by the Conjoint Board of England as a place where undergraduates may spend six months of their fifth year in clinical work. There are 210 beds, to which nearly 3,000 cases are admitted per annum, and in the out-patient department the average number of new cases each year exceeds 24,000. Clinical instruction is given daily in the wards, out-patient department, and operating theatre by members of the visiting staff. Fees for one month's attendance, two guineas; for three months, five guineas; perpetual ticket, ten guineas; clinical clerk's reduced fee of £1 1s. for three months. A certificate is given at the end of a three months' course. Further details and full syllabus may be obtained by application to the Dean or the Secretary of the hospital. A limited number of clerkships in the pathological department, affording facilities for theoretical and practical instruction in clinical pathology and bacteriology suitable for post-graduates and fifth-year students, are available. Fees for one month's course, three guineas; two months' course, five guineas; three months' course, six guineas. For detailed syllabus application should be made to the Dean or the Secretary. The Dean is Mr. George Waugh, from whom all further information can be obtained, or from the Secretary at the hospital.

